# Radiological risk factors for T2-T3 glottic carcinoma treated with (chemo)radiotherapy—a multicenter analysis

**DOI:** 10.1007/s00330-026-12704-3

**Published:** 2026-06-19

**Authors:** Agatha S. Baidun, Yves M. H. F. Gorissen, Martine Hendriksma, Renske Gahrmann, Jose A. Hardillo, Sjoert Pegge, Robert P. Takes, Guido B. van den Broek, Martin A. de Jong, Federica Lucev, Elisabeth V. Sjogren, Berit M. Verbist

**Affiliations:** 1 Department of Otorhinolaryngology and Head and Neck Surgery, Leiden University Medical Center, Leiden, The Netherlands; 2 Department of Radiology and Nuclear Medicine, Erasmus Medical Center, Rotterdam, The Netherlands; 3 Department of Otorhinolaryngology and Head and Neck Surgery, Erasmus Medical Center, Rotterdam, The Netherlands; 4 Department of Radiology, Radboud University Medical Center, Nijmegen, The Netherlands; 5 Department of Otorhinolaryngology and Head and Neck Surgery, Radboud University Medical Center, Nijmegen, The Netherlands; 6 Department of Radiation Oncology, Leiden University Medical Center, Leiden, The Netherlands; 7 Department of Radiology, Fondazione I.R.C.C.S. Policlinico San Matteo, Pavia, Italy; 8 Department of Radiology, Leiden University Medical Center, Leiden, The Netherlands

**Keywords:** Laryngeal neoplasms, Radiotherapy, Risk factors, Tomography, X-ray computed, Magnetic resonance imaging

## Abstract

**Objectives:**

In moderately advanced (T2-T3) glottic cancer, eligibility for organ-preserving surgery is limited by tumor extension into certain high-risk areas within the larynx that can be identified on preoperative imaging and serve as predictive factors for poorer outcomes. Their relevance in conservative treatment modalities, including (chemo)radiotherapy ([C]RT), remains unclear. The objective was to evaluate the impact of these predefined surgical high-risk zones in patients treated with (C)RT.

**Materials and methods:**

This retrospective study included patients with T2-3, N0-3, M0 glottic carcinoma treated with primary (C)RT and with available diagnostic imaging from three academic medical centers between 2014 and 2018. Imaging was scored for tumor involvement of high-risk areas. The 5-year rates of locoregional control (LRC) and disease-specific survival (DSS) were compared using the log-rank test and Cox proportional hazards model.

**Results:**

Imaging from 180 patients (mean age, 68 ± 11 years, 154 men) was assessed, with 173 entering the risk factor analysis after excluding unreliable scans. Significant findings were observed in the anterior larynx: vertical anterior commissure involvement negatively affected LRC and DSS, while horizontal anterior commissure and anterior paraglottic space reduced LRC. Deep vocal muscle infiltration showed negative trends. In multivariable analysis, vertical anterior commissure infiltration retained a trend toward lower DSS.

**Conclusion:**

Involvement of the anterior commissure, particularly vertical extension, which is a known predictive factor from surgical studies, likely predicts poorer outcomes after (C)RT for T2-T3 glottic carcinoma. Involvement of the anterior paraglottic space and depth of tumor infiltration carry potential predictive value.

**Key Points:**

***Question*** *Can radiological risk factors proven in surgically treated patients with T2-T3 glottic carcinoma also predict treatment failure in patients treated with (chemo)radiotherapy?*

***Findings*** *On multivariable analysis, vertical anterior commissure involvement showed a trend toward poorer disease-specific survival.*

***Clinical relevance*** *Our findings contribute to the search for harmonized prognostic factors across treatment modalities for glottic carcinoma and provide a basis for integrating these risk factors into staging and treatment counseling.*

**Graphical Abstract:**

**Figure:**
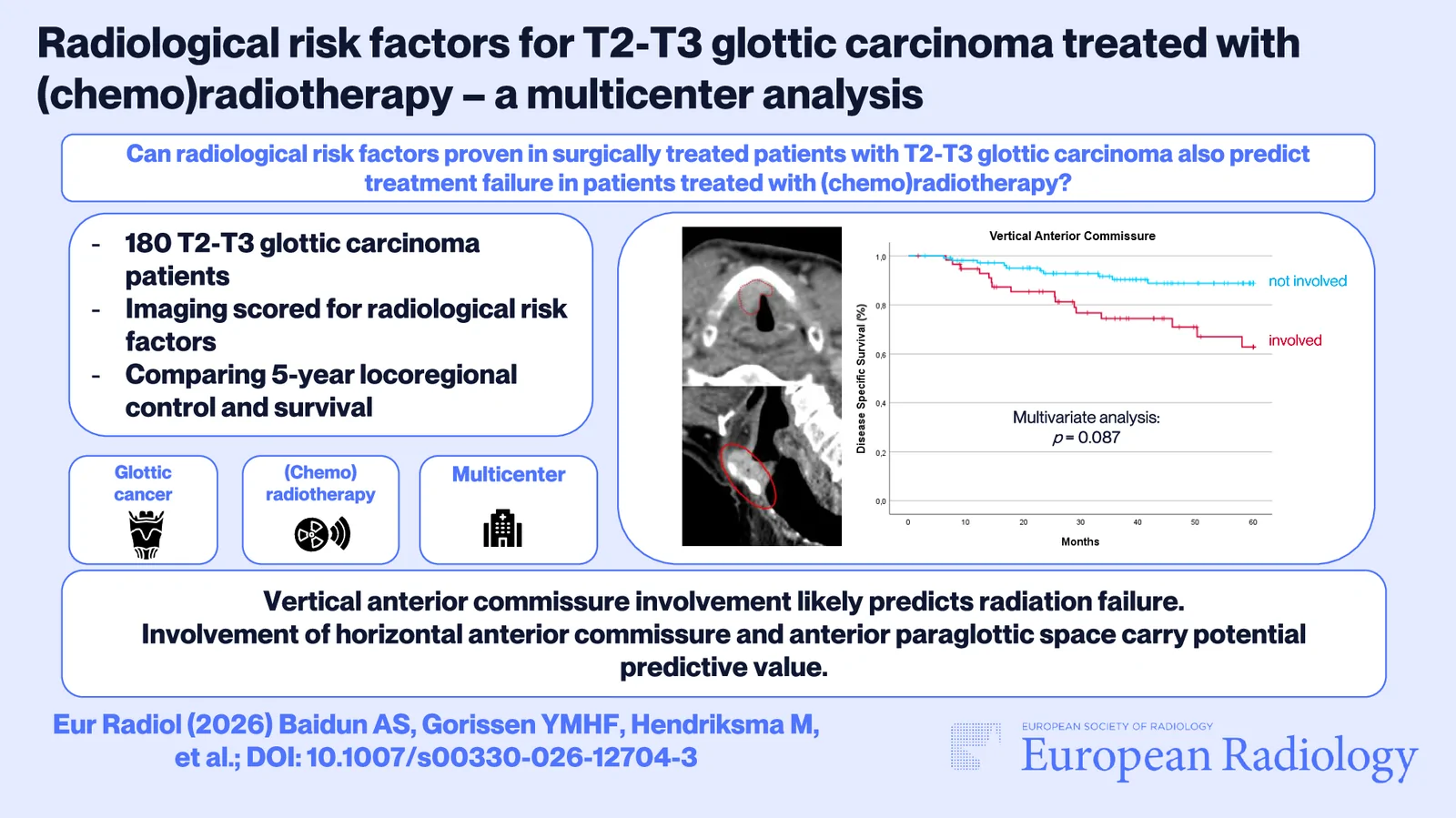

## Introduction

The larynx is an anatomically intricate organ where primary tumors exhibit varying patterns of spread, significantly impacting prognosis. With increasing use of laryngeal preservation strategies such as (chemo)radiotherapy ([C]RT), transoral CO_2_ laser microsurgery (TOLMS), and, less commonly, open partial laryngectomy (OPL), careful patient selection has become essential to ensure effective tumor control while maintaining organ function.

TOLMS has gained prominence in recent decades, with indications expanding from early to moderately advanced glottic cancer. Numerous studies demonstrate its effectiveness in T2 and selected T3 cases, showing oncologic results comparable or superior to RT or open surgery [1–3]. However, not all patients within these stages are suitable candidates, as several studies have identified high-risk zones where incomplete resection and tumor recurrence are more likely after surgical treatment, including the anterior commissure (AC), posterior visceral spaces, and cartilaginous framework [4–9]. Patients with such tumor extensions are usually ineligible for TOLMS and are instead offered OPL or (C)RT. Yet, tumors treated conservatively with (C)RT have rarely been stratified by the criteria recommended for surgical cases [7, 10], and most relevant studies date back several years [11–16]. A recent study by Hendriksma et al was the first to identify vertical AC involvement, a known surgical risk factor, as a predictive factor for radiation failure, while horizontal AC involvement and deep vocal muscle infiltration showed non-significant trends toward poorer outcomes [17].

Although (C)RT is an effective treatment option, it carries risks of severe late toxicities and higher laryngectomy rates due to limited salvage options [18]. Also, the untested assumption that patients with surgical risk factors on imaging will do better with conservative treatment may be incorrect. Therefore, understanding tumor behavior and recurrence patterns across both surgical and non-surgical approaches is essential for adequate treatment comparison and patient counseling. As such, it must be clearly determined whether (C)RT truly offers better oncological outcomes than TOLMS or OPL for tumors in these specified sub-localizations. Moreover, if certain tumor growth patterns within the T2-T3 stages consistently result in poorer outcomes regardless of treatment, it may affirm inconsistencies in the current TNM system and significantly impact treatment and follow-up strategies. To address this gap, this study investigates surgical high-risk zones within a (C)RT cohort to identify predictors of radiation failure.

## Materials and methods

### Patient selection and data extraction

**Fig. 1:**
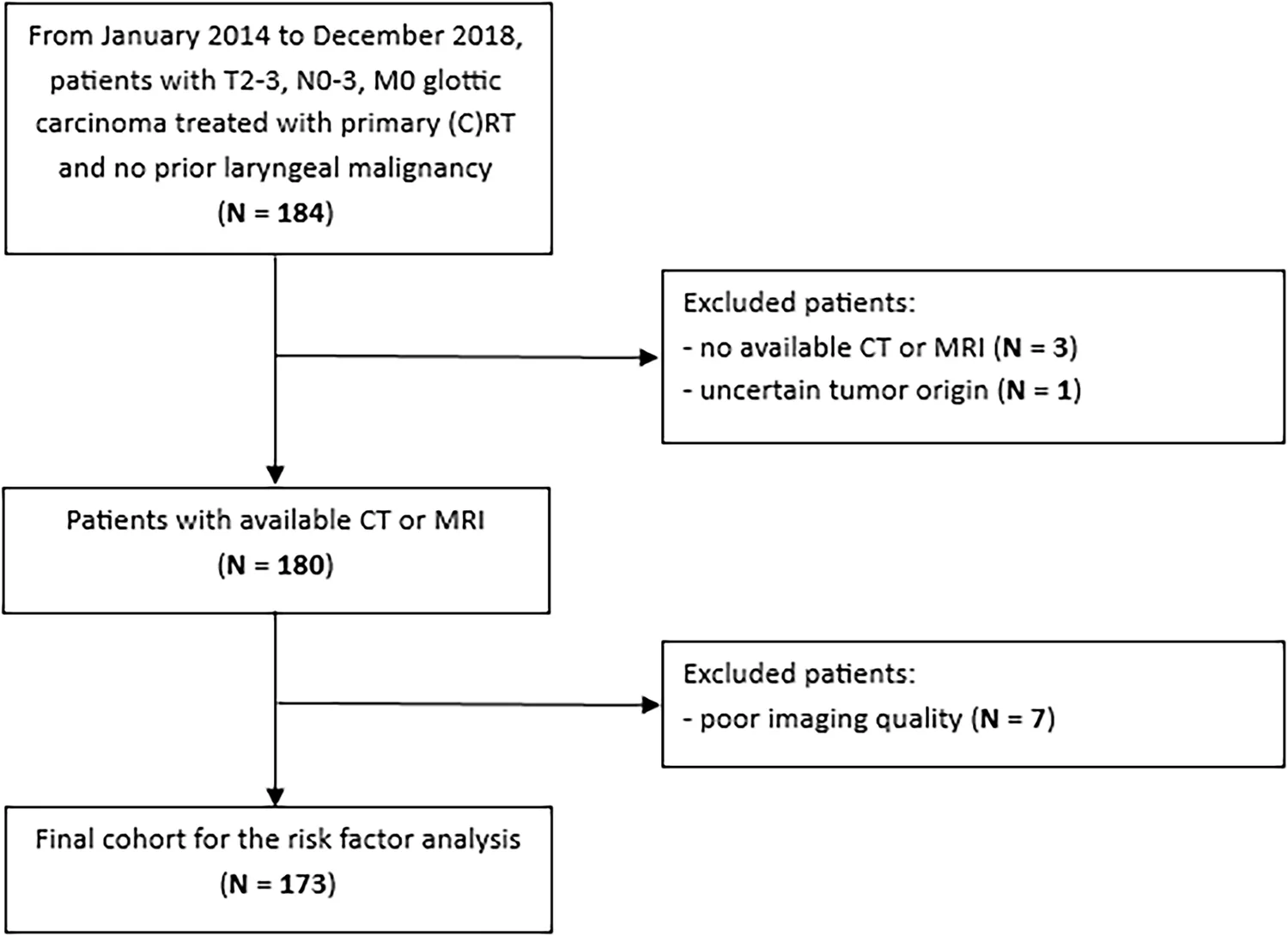
Study flowchart. (C)RT, (chemo)radiotherapy

The study adhered to the Declaration of Helsinki and received approval from the Leiden University Medical Center ethics committee on 10 October 2022 (ref. 22-3050). Consecutive patients were retrospectively included from three Dutch academic centers: Leiden University Medical Center, Erasmus Medical Center, and Radboud University Medical Center. Inclusion criteria were T2-3, N0-3, M0 glottic carcinoma treated with primary (C)RT between January 2014 and December 2018, available pre-treatment CT or MRI, and no prior laryngeal malignancy (Fig. 1). Tumors were staged at diagnosis using the 7th or 8th AJCC/UICC TNM edition, as the transition occurred during the study period. Data extracted from electronic medical records included demographics, radiological tumor characteristics, RT technique, treatment details, adverse events (scored according to the Common Terminology Criteria for Adverse Events [19]), follow-up, and oncological outcomes.

Radiotherapy consisted of 60 Gy in 25 fractions of 2.4 Gy, or 70 Gy in 35 fractions of 2 Gy to the primary tumor and/or involved lymph nodes; elective nodal irradiation was institution-based. Patients aged ≤ 70 years were generally treated with 6 fractions per week, whereas older patients or those receiving concurrent chemotherapy received 5 fractions per week. Contrast-enhanced planning CT was used for target delineation. For the larynx only, we defined the entire larynx to be irradiated in that time frame; bolus was used when the AC was involved. In case of locoregional RT, gross tumor volume was defined on the planning CT with the use of clinical, MRI, and/or PET-CT information. Clinical target volume margins were 1 cm/0.5 cm for tumor and/or involved nodes, edited to their respective anatomical barriers. Planning target volume margins were 0.5 cm mostly. Cone beam CT-guided online registration was performed daily pre-treatment.

**Fig. 2:**
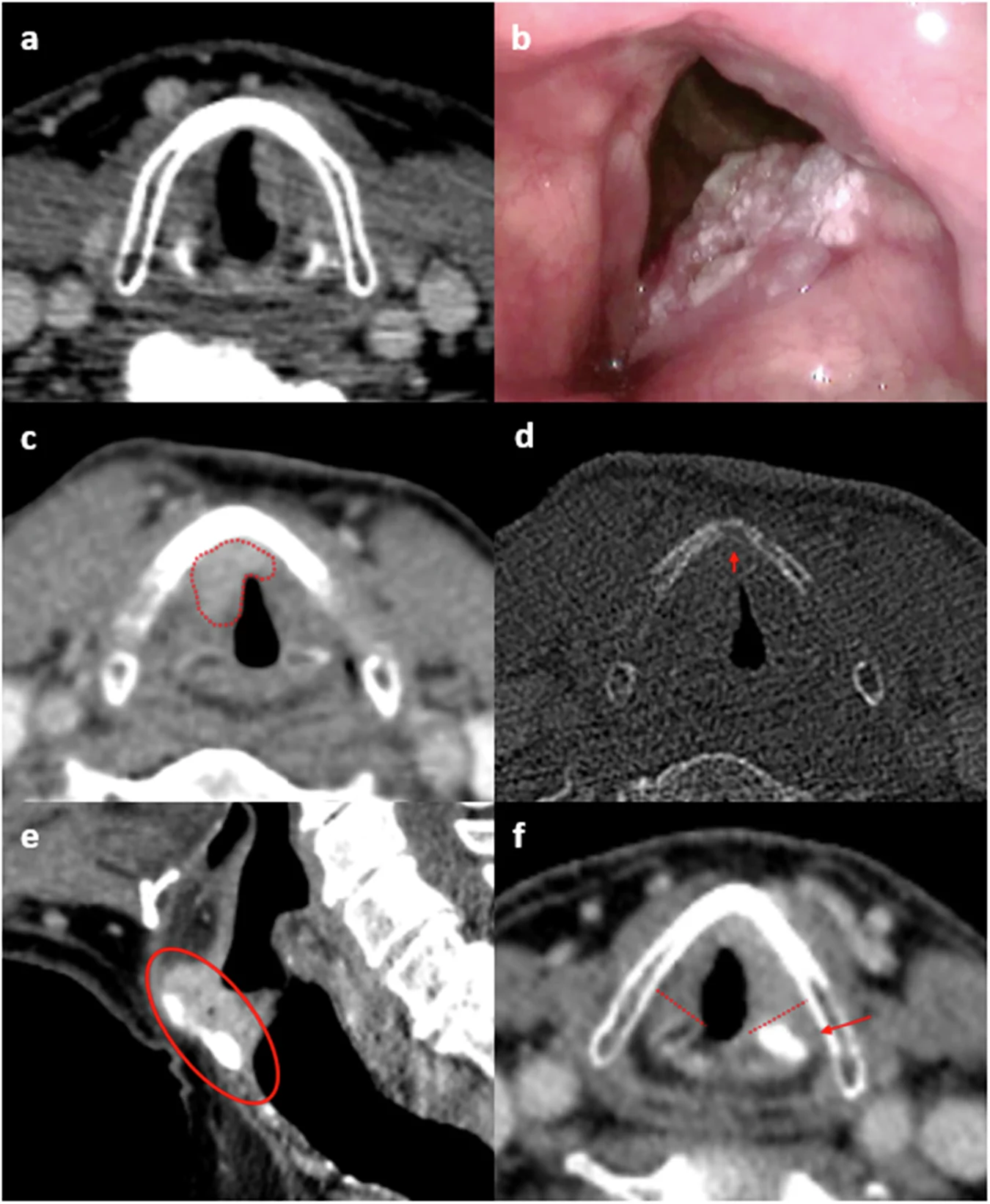
Examples of different patterns of tumor spread. **a** Superficial tumor of the left vocal fold not infiltrating into the predefined high-risk zones; **b** corresponding endoscopic examination of the superficial tumor; **c** deep infiltration into the anterior half of the right vocal muscle and involvement of the anterior paraglottic space, horizontal involvement of the anterior commissure (dotted line); **d** cortical erosion of the thyroid cartilage at the level of the anterior commissure (arrow); **e** vertical involvement of the anterior commissure (circle); **f** normal hypodense paraglottic fat behind the M-line (dotted line) visible on the right is obliterated by tumor on the left (arrow)

For radiological characteristics, imaging was reviewed by three experienced head-and-neck radiologists (R.G., S.P., F.L.), blinded to outcomes, all trained on the assessment protocol by the leading radiologist (B.M.V.). Assessed variables included: (1) superficial versus deep vocal fold muscle infiltration, based on whether the tumor extends beyond one-third of the thyroarytenoid muscle; (2) paraglottic space (PGS) involvement, either anterior or posterior to the M-line, defined by Succo et al as a line tangential to the arytenoid vocal process and perpendicular to the ipsilateral thyroid lamina [20]; (3) thyroid cartilage infiltration; (4) horizontal or vertical AC involvement, based on confinement to the glottic plane or showing supraglottic/subglottic extension; according to standard radiological definitions, the glottis extends form the apex of the laryngeal ventricle to 1 cm below the upper vocal cord surface, with regions above and below considered supraglottic and subglottic, respectively; and (5) tumor volume in mL, categorized as < 3.5 mL or ≥ 3.5 mL based on the Dutch guideline threshold for additional chemotherapy [21] (Fig. 2). Tumor characteristics were assessed separately for left and right sides; in cases of bilateral tumors, the more severe findings from either side were recorded. Tumors not visible on imaging of sufficient quality were classified as superficial, without PGS or thyroid cartilage involvement; AC involvement could not be reliably scored in these cases and was excluded from the corresponding analysis.

Patients were followed in accordance with national guidelines, which recommend a follow-up period of 3–5 years, with clinical examinations every 2–3 months during the first year, gradually decreasing to every 6 months at 5 years [22].

### Statistical analysis

Data were analyzed using SPSS version 29.0. Outcomes included 5-year locoregional control (LRC), laryngeal preservation (LP), disease-specific survival (DSS), and overall survival (OS). The starting point for all time-to-event analyses was RT initiation. Endpoints were defined as the date of first recurrence for LRC, the date of total laryngectomy for LP, the date of death from laryngeal cancer for DSS, and the date of death from any cause for OS. Median follow-up was calculated using the reverse Kaplan–Meier method. For the risk factor analysis, patients were excluded if tumor growth patterns could not be reliably assessed due to insufficient imaging quality. Potential radiological prognostic factors, limited to those most relevant to this study, were assessed for LRC and DSS using univariate log-rank tests. Multivariate analysis was performed on (marginally) significant factors using the Cox proportional hazards model.

## Results

### Patients and follow-up

Between 2014 and 2018, 180 patients with T2-3, N0-3, M0 glottic carcinoma met the inclusion criteria. CT was the most common imaging modality (*n* = 178), while only two cases had MRI as the sole available modality. Only four patients received adjuvant chemotherapy, of which three had cisplatin and one had cetuximab. Further patient and treatment characteristics are presented in Table 1. The median follow-up of the entire cohort was 62.0 months (95% CI: 59.1–65.0), ranging from 2 to 115 months. Fifty-six patients (31.1%) had follow-up durations shorter than our 5-year endpoint. Documented reasons included transfer of care to another hospital or relocation abroad (*n* = 7), advanced age or declining health (*n* = 4), and COVID-19-related disruption (*n* = 2). The remaining cases (*n* = 43) had no recorded reason; this group also includes patients with a 3-year follow-up consistent with current guidelines [22]. The median follow-up in the lost group was 37.0 months (95% CI: 33.7–40.3).

**Table 1:** Patient and treatment characteristics

	Number of patients (%) Total = 180 (100%)
Patient characteristics	
Center	
LUMC	37 (20.6)
EMC	75 (41.7)
Radboud UMC	68 (37.8)
Sex	
Male	154 (85.6)
Female	26 (14.4)
Mean age (SD)	67.8 (10.5)
ASA score	
1	14 (7.8)
2	122 (67.8)
3	43 (23.9)
4	1 (0.6)
T stage	
T2	117 (65.0)
T3	63 (35.0)
N stage	
N0	173 (96.1)
N1	6 (3.3)
N2a	0 (0)
N2b	1 (0.6)
Treatment characteristics	
Treatment	
RT	176 (97.8)
CRT	4 (2.2)
RT technique	
IMRT	94 (52.2)
VMAT	60 (33.3)
3D conformal RT	20 (11.1)
Not specified	6 (3.3)
Median RT total dose tumor in Gy (range)	68.0 (38.0–70.0)*
Median RT fraction dose tumor in Gy (range)	2.0 (2.0–4.0)*
Cervical lymph node RT	
Yes (N0)	109 (60.6)
Yes (N+)	7 (3.9)
No	64 (35.6)
Median RT dose neck in Gy (range)	54.3 (29.5–67.5)*

*LUMC* Leiden University Medical Center, *EMC* Erasmus Medical Center, *Radboud UMC* Radboud University Medical Center, *SD* standard deviation, *RT* radiotherapy, *CRT* chemoradiotherapy, *IMRT* intensity-modulated radiotherapy, *VMAT* volumetric modulated arc therapy, *Gy* gray

* Two patients received non-standard radiotherapy due to treatment interruption or initially palliative intent with subsequent complete response, accounting for the observed variation in total dose and fraction size; all other patients were treated according to protocol

### Oncological outcomes

During follow-up, 45 patients (25.0%) presented with locoregional recurrences after a median of 11.3 months (range 4–55 months), and 86.7% were detected within the first 2 years after treatment. The 5-year LRC rate was 71.4%. Of the eight patients (4.4%) with regional recurrence, five received neck irradiation during primary treatment and three did not.

Following recurrent disease, total laryngectomy was performed in 20 patients (11.1%). The 5-year LP rate was 86.7%. Five patients underwent alternative surgical salvage: one had TOLMS after refusing total laryngectomy; one had partial laryngectomy for subglottic recurrence; and three had cervical lymph node dissection for isolated regional disease, with one also receiving additional neck irradiation. In five cases, total laryngectomy remained a curative option, but patients declined treatment and received best supportive care. Another 15 patients were given palliative care. One patient relocated abroad, and their subsequent management is unknown.

At 5 years, 28 patients (15.6%) died of glottic cancer and 48 patients (26.7%) of unrelated causes, resulting in 5-year DSS and OS rates of 80.9% and 56.9%, respectively.

### Risk factor analysis

Diagnostic imaging was available for all 180 patients. For the risk factor analysis, seven patients were excluded due to poor imaging quality caused by motion artifacts or suboptimal contrast timing, leaving 173 patients in the final analysis. In some cases, radiologists reported uncertainty regarding tumor infiltration into certain areas; these uncertain groups were too small for meaningful separate comparison and were therefore excluded from the corresponding analysis. For the same reason, stratification of tumor volume was not performed, as only 6.9% of patients had tumors ≥ 3.5 mL.

**Fig. 3:**
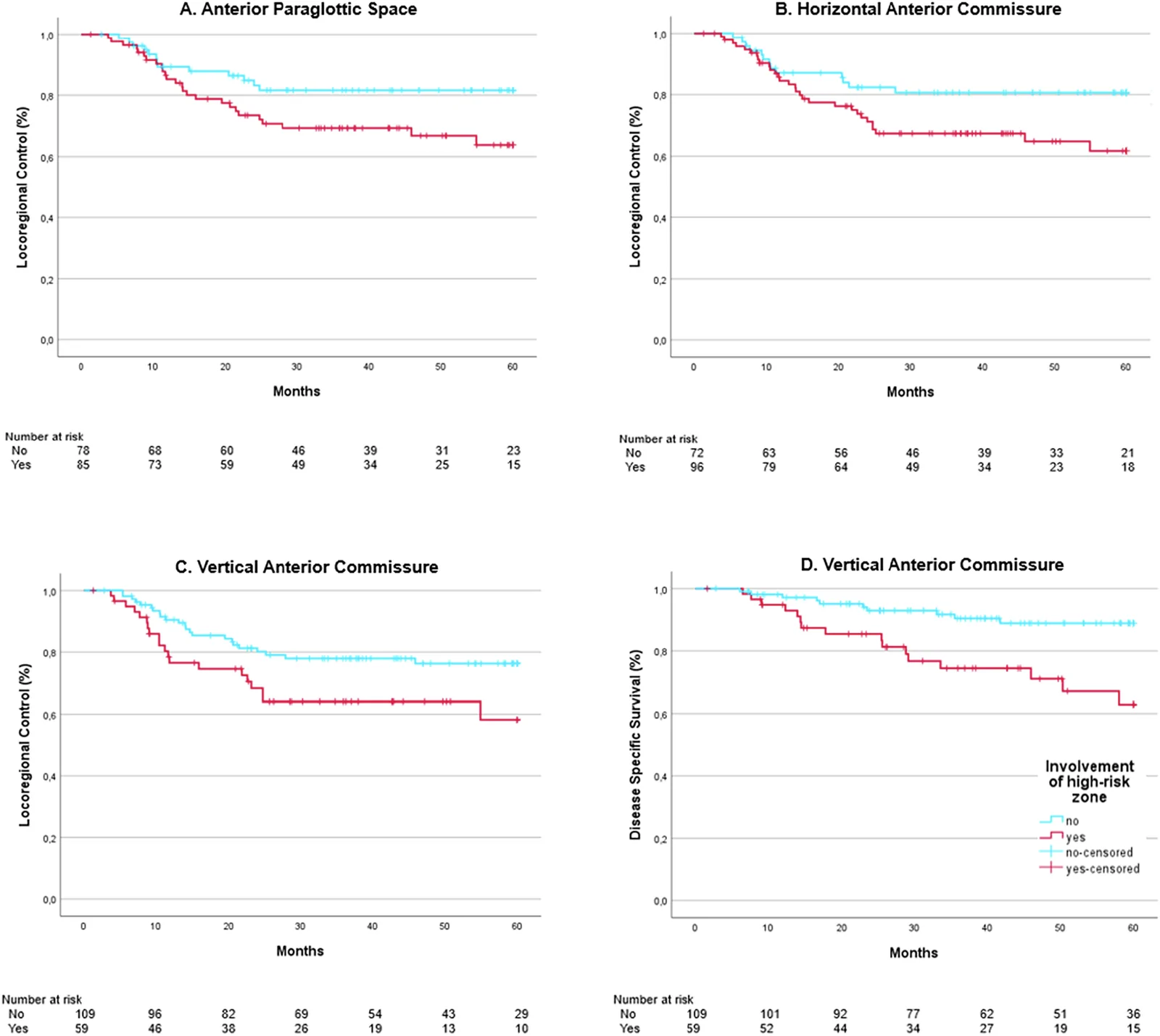
Outcome stratification for significant high-risk zones. Locoregional control stratified for **a** anterior paraglottic space, **b** horizontal anterior commissure, **c** vertical anterior commissure, and **d** disease-specific survival stratified for vertical anterior commissure

In univariate analysis, vertical AC involvement was significantly associated with reduced LRC and DSS, and involvement of both horizontal AC and anterior PGS significantly reduced LRC (Table 2, Fig. 3). No other statistically significant risk factors were identified, although deeper vocal fold infiltration showed a trend toward worse outcomes. In multivariable analysis, no variables retained statistical significance, although vertical AC involvement continued to demonstrate a trend toward lower DSS (Table 3).

**Table 2:** Univariate risk factor analysis at 5 years

Radiological characteristics	No of patients (%) Total = 173 (100%)	5-year LRC (%)	*p*-value	5-year DSS (%)	*p*-value
Vocal fold muscle infiltration					
Superficial (< 1/3)	58 (33.5)	81.5	0.080	88.9	0.081
Deep (> 1/3)	115 (66.5)	65.5		76.1	
Infiltration PGS anterior					
No	78 (45.1)	81.6	0.047*	85.2	0.398
Yes	85 (49.1)	63.8		78.3	
Uncertain	10 (5.8)				
Infiltration PGS posterior					
No	135 (78.0)	69.0	0.360	81.0	0.586
Yes	34 (19.7)	79.2		74.1	
Uncertain	4 (2.3)				
Infiltration thyroid cartilage					
No	140 (80.9)	70.5	0.310	79.3	0.237
Yes	20 (11.6)	84.4		94.1	
Uncertain	13 (7.5)				
Horizontal involvement AC^a^					
No	72 (41.6)	80.6	0.043*	88.6	0.066
Yes	96 (55.5)	61.7		73.0	
Vertical involvement AC^a^					
No	109 (63.0)	76.3	0.037*	88.9	0.001*
Yes	59 (34.1)	58.2		62.9	

*LRC* locoregional control, *DSS* disease-specific survival, *PGS* paraglottic space, *AC* anterior commissure

* Significant values (*p* < 0.05)

^a^ In five patients, the tumor was not visible despite adequate imaging, preventing reliable classification of AC involvement, as described in the “Methods” section

**Table 3:** Multivariate analysis at 5 years in hazard ratios

Characteristics	5-year LRC (95% CI)	*p*-value	5-year DSS (95% CI)	*p*-value
Vocal fold muscle infiltration				
Superficial (< 1/3)				
Deep (> 1/3)	1.108 (0.363–3.381)	0.857	2.006 (0.503–8.005)	0.324
Infiltration PGS anterior				
No				
Yes	1.569 (0.603–4.080)	0.356	0.712 (0.235–2.160)	0.549
Horizontal involvement AC				
No				
Yes	1.566 (0.693–3.538)	0.280	1.173 (0.387–3.561)	0.778
Vertical involvement AC				
No				
Yes	1.296 (0.615–2.732)	0.495	2.484 (0.877–7.036)	0.087

*LRC* locoregional control, *DSS* disease-specific survival, *CI* confidence interval, *PGS* paraglottic space, *AC* anterior commissure

### Toxicity

No patients died from RT-related toxicity. Grade 4 toxicity occurred in three patients (1.7%), all requiring emergency tracheostomy, with one ultimately progressing to a total laryngectomy due to a non-functional larynx. Grade 3 toxicity occurred in 20 patients (11.1%): 18 required nasogastric feeding for dysphagia; one was hospitalized with laryngeal edema and aspiration pneumonia requiring antibiotics; and one experienced recurrent laryngeal edema necessitating non-emergency tracheostomy. Two patients developed subclinical hypothyroidism requiring hormone replacement (grade 2). Among the four CRT patients, one developed nephrotoxicity requiring dialysis (grade 3).

## Discussion

### Treatment results

This study assessed the efficacy of (C)RT in patients with tumor extension in well-known surgical risk areas in T2-T3 glottic carcinomas. As only four patients (2.2%) received CRT, no definite conclusions can be drawn regarding the effect of concurrent chemotherapy. Our treatment resulted in 5-year outcome rates of 71.4% for LRC, 86.7% for LP, 80.9% for DSS, and 56.9% for OS, most of which fall within the previously reported—albeit wide—ranges in the literature on (C)RT. Only LP was slightly better than expected [1, 3, 18, 23–26].

The main objective of this study was to identify predictors of radiation failure by stratifying patients according to the high-risk zones previously identified for surgery (TOLMS/OPL). With this approach, we found that involvement of the AC and anterior PGS was associated with poorer outcomes in univariate analysis, while vertical AC showed a persistent trend toward reduced DSS in the multivariate model. To our knowledge, this is the largest cohort in the past two decades evaluating prognostic factors for radiation failure in moderately advanced glottic cancer. Our findings align with previous, though limited, reports and further point to the likely relevance of these zones—particularly vertical AC involvement—in RT treatment, highlighting the need to include these factors in future studies. The absence of independent prognostic factors in multivariable analysis may at first consideration contradict their relevance. However, we would argue that it can also reflect overlap in anatomic areas and the challenges in distinguishing them on CT imaging, especially considering interobserver variability. Additionally, the wide 95% confidence intervals suggest that our sample size may still be insufficient. Therefore, in our opinion, the “zero-sum” result of the multivariable analysis does not disqualify the potential clinical relevance of the evaluated parameters, although a certain degree of caution must be maintained.

Given the scarcity of research in this area, we aimed to summarize existing literature and contextualize our findings in the rest of the “Discussion” to provide a better understanding of these high-risk zones and offer guidance on their application in staging and treatment.

### Infiltration into the anterior commissure and thyroid cartilage

The AC can be considered a distinct anatomical entity within the larynx, more susceptible to tumor progression due to the narrow distance between mucosa and cartilage, the absence of an inner perichondrium, and its proximity to the visceral spaces and cricothyroid membrane [27–30]. Additionally, microinfiltration of the thyroid cartilage is shown to be more common than would be suspected on routine clinical work-up [31–35], with Yener et al reporting radiologically undetected cartilage erosion in 22% of cases [36]. This anterior area poses technical challenges in treatment, as it is often difficult to expose during TOLMS and prone to underdosage during RT [37, 38]. At first glance, literature regarding the impact of AC involvement appears conflicting for both treatment modalities. In a systematic review, Hendriksma et al found that 32% of RT studies and 25% of TOLMS studies reported a negative impact of AC involvement, while the remainder did not [17]. Nonetheless, pooled analysis showed superior 5-year local control (LC) in AC-negative tumors across both treatment modalities, with differences of 8.6% for RT and 13.8% for TOLMS. This discrepancy was attributed to variations in AC staging, and recommendations for a distinction between horizontal and vertical AC involvement instead of a simple binary approach (yes/no) were reiterated from previous publications [7, 39, 40]. Notably, when such stratification is implemented in surgical series, the negative impact of AC involvement, especially vertical extension, becomes evident [6, 41, 42]. To our knowledge, only one smaller study, performed at one of the participating institutions, has stratified patients treated with RT accordingly and found vertical AC involvement to be predictive of radiation failure, while horizontal involvement showed a non-significant trend [23]. Combined with our current multicenter findings, this suggests that AC involvement, especially vertical extension, may lead to worse outcomes for both TOLMS and (C)RT, and should therefore be considered a common prognostic factor in surgical and conservative treatment.

As both TOLMS and (C)RT likely have reduced efficacy in tumors extending vertically over the AC, alternative treatment modalities should be considered. In theory, if the complex anatomy of the AC and/or thyroid cartilage infiltration contributes to poorer outcomes, open surgical approaches, where the thyroid cartilage can be resected en bloc, should yield better results. Indeed, the prognostic gap of AC involvement disappears when using OPL techniques, restoring LC rates of AC-positive tumors to those without AC involvement [43–45]. Therefore, to achieve surgical radicality, especially in gross AC involvement or in salvage cases, OPL may need to be considered more frequently. Regarding the addition of chemotherapy, our study is inconclusive as only four patients received CRT. Furthermore, evidence from the literature for this patient population is limited and not stratified for AC involvement. Two meta-analyses on T3 laryngeal cancer found CRT to be comparable to total laryngectomy and superior to RT for survival and LC, though both noted significant heterogeneity in CRT studies, and RT outcomes were considerably worse than ours (OS 40.8–53.2%; LC 50.4%; LP 43.9%) [3, 46]. Thus, the benefit of additional chemotherapy is still to be determined. However, ongoing developments in RT techniques may enhance its potential as a single-modality non-surgical alternative for anterior tumors.

### Infiltration into the vocal muscle and paraglottic spaces

For the depth of tumor infiltration, current TNM staging relies on vocal fold mobility, with reduction indicating significant extension into the thyroarytenoid muscle [47, 48]. Using hypomobility as a surrogate, numerous studies have linked deeper infiltration with worse prognosis for both TOLMS and RT, with approximately a 10% difference in 5-year LC rates [1, 18]. We observed a non-significant trend toward poorer outcomes in deeply infiltrative tumors on imaging, similar to a previous study by Hendriksma et al [23]. Therefore, the depth of infiltration warrants further investigation and could potentially serve as a more reliable alternative to the subjective mobility criterion.

PGS infiltration is generally considered a risk factor for treatment failure, hence the upstaging from T2 to T3 in these cases. In surgical patients, posterior extension into the thyrocricoarytenoid space—defined by Succo et al as infiltration beyond the M-line [20]—is particularly disadvantageous as this involves a region rich in vessels and lymphatics with only limited separation from hypopharyngeal mucosa and the open neck. Surgical treatment of these posterior lesions is less effective than that of tumors involving the anterior PGS [49, 50]. Accordingly, Succo et al demonstrated that in anterior T3 tumors, OPL achieves excellent radicality and outperforms both TOLMS and CRT [20]. For posterior tumors, however, even OPL appears insufficient, and the authors suggest that these tumors are more comparable to T4 lesions requiring total laryngectomy. The effectiveness of RT in PGS involvement remains unclear. A meta-analysis found moderate support for PGS involvement as a prognostic factor on CT, but this was based on four studies performed 20 years ago, none of which distinguished anterior from posterior PGS [51]. Contrary to earlier surgical studies, we found anterior PGS involvement to result in decreased LRC in univariable but not in multivariable analysis. This could reflect the limited impact of this area in (C)RT, though it could also indicate challenges in delineating the narrow anterior PGS on imaging. Posterior PGS involvement showed no negative impact, which may be caused by the lack of power. Alternatively, it remains possible that posterior PGS involvement may simply not represent a significant risk factor for radiation failure and that this is the preferential treatment for these tumors.

### Limitations

Limitations include the variable follow-up duration across centers, limiting our ability to reach the 5-year endpoint for all patients. In line with the option offered in Dutch guidelines [22], one center routinely followed patients for 3 years, while the other two extended follow-up to 5 years. Nevertheless, all centers captured the critical period, as most recurrences occur within the first 2 years. Furthermore, interpretation of the scans was performed by three radiologists, each reviewing imaging from their respective centers, where differences in acquisition techniques and protocols may have influenced interpretation. Although interobserver variability cannot be excluded, all reviewers were dedicated head-and-neck radiologists trained on the image assessment protocol by the leading radiologist. Lastly, despite being multicenter and the largest to date, our study may be underpowered as suggested by the wide confidence intervals. Excluding uncertain cases from the risk factor analysis, though methodologically necessary, further reduced the number of events. As the number of patients and events dictates how many factors can be included without losing statistical power, we could not assess all potential predictors (e.g., RT dose, technique, adjuvant chemotherapy), though this does not rule out their influence. Also, while the effects observed in certain high-risk zones indicate potential clinical relevance, they require confirmation in larger, prospective studies where the above-mentioned biases can be further reduced.

## Conclusion

This study shows that known surgical predictive factors related to tumor extension into certain high-risk areas within the larynx, particularly vertical AC involvement, are likely associated with poorer locoregional control and survival, also in patients treated conservatively with (C)RT. Furthermore, involvement of the anterior PGS and depth of tumor infiltration carry potential predictive value but require further validation. These findings contribute to the search for harmonized prognostic factors across treatment modalities and provide a basis for integrating these risk factors into staging and treatment counseling.

## Abbreviations

AC: Anterior commissure
(C)RT: (Chemo)radiotherapy
DSS: Disease-specific survival
LC: Local control
LP: Laryngeal preservation
LRC: Locoregional control
OPL: Open partial laryngectomy
OS: Overall survival
PGS: Paraglottic space
TOLMS: Transoral CO_2_ laser microsurgery
